# Improved Operation for Staphyloraphy

**Published:** 1826-07

**Authors:** 


IMPROVEMENT IN THE GAUMENNATH* OPERATION, OR STAPHYLORAPHY.

The two difficult points in this operation are first, the slicing off the edges of the divided palate, secondly, the introduction of the sutures, and thirdly the difficulty of making the ligatures sufficiently secure when introduced. In the February Number of HECKUS *Annalen*, Dr. DIEFFENBACK has given a description of some instru-

---

* We want an English word by which to call this operation. As the operation is originally German, we have retained here the term Gaumennath, from *Gaumen* the palate, and *Nath* a suture, but this is not exactly the thing we want; we should not feel contented to call the operation in English the *Palatesuture*. Mr. Roux's name of staphyloraphy is not strictly correct, since ϛαφυλή means rather the *uvula* than the palate: Although it is evident that we should be justified by the precedent just stated in calling the operation the Palatesuture.—*Rev.*

ments which tend to lessen very much these difficulties. For the more ready excision of the edges, he proposes that a somewhat curved forceps should be used with their narrow blades the length of the usual division of the palate, and turned at nearly right angles with the arms of the instrument. With this forceps the edge of the palate is so taken hold of, and held sufficiently firm to allow of the slicing off of the edge of the palate projecting beyond the blades of the forceps, which is then to be carried a little farther back, and used as a compress to suppress the hæmorrhage during the introduction of the needles. These *needles* are only seven lines long, from the point to their middle a little curved and cutting on their edges, with their concave surface flat. The other half of the needle is perfectly round and hollow, into which extremity is worked a fine mother-screw. The ligature is made of *fine lead wire*, corresponding to the size of the round extremity of the needle, and capable of being worked into the screw contained in it. The advantages afforded by the use of this material for ligature are several; it allows of being tightened or loosened at pleasure, it allows of time during the operation of examining the mouth without becoming soft and almost unmanageable as the common ligature is when wetted, for with a scissors the end of the lead ligature may be cut off near the palate, the end twisted toward the roof of the mouth, and allowed to remain without inconvenience for a short time. The ends of the ligature are twisted slowly together after the approximation of the velum, and thus make the parts firm and secure. If, from a high degree of inflammation supervening, the swelling should be such as to require the ligatures to be loosened a little, it can be easily done, by untwisting the ends of the lead-wire partially. The chance of success is with this apparatus greatly increased.

An instrument has lately been invented by Dr. Dieffenback of Berlin, for the division of strictures from within the urethra, which cuts from behind forwards. It is a concealed knife, having three blades enclosed in a small cylinder, and separated by touching a spring left in the handle. The cylinder containing the cutting instrument is passed through another and graduated catheter to the stricture, it is then pushed forwards through the stricture, and when the extremity reaches beyond it, the blades are opened, and the stricture divided as the small cylinder is withdrawn toward the lower orifice of the greater, and then the blades are again shut. Dr. D. says that it is a quick, safe, and effectual manner of curing old strictures, when gradual pressure is afterwards employed.

